# Effects of Low-Level Laser Therapy on Oral Mucosal Wound Healing and Systemic Oxidative Stress in Diabetic Rats: An In Vivo Experimental Study

**DOI:** 10.3390/medicina61091651

**Published:** 2025-09-11

**Authors:** Nadica S. Đorđević, Ilija M. Dragojević, Aleksandra N. Ilić, Nikola M. Stojanović, Jelena T. Todić, Dragana Puhalo Sladoje, Ivana Stošović Kalezić, Aleksandar M. Đorđević, Radovan Jovanović, Ljiljana Šubarić, Gordana Filipović, Zdenka Stojanović, Milena Kostić

**Affiliations:** 1 Department of Dentistry, Faculty of Medicine, University of Priština in Kosovska Mitrovica, 38220 Kosovska Mitrovica, Serbia; jelena.todic@med.pr.ac.rs (J.T.T.); ivana.stosovic@med.pr.ac.rs (I.S.K.); aleksandar.djordjevic@med.pr.ac.rs (A.M.Đ.); radovan_jovanovic@hotmail.com (R.J.); emajel@gmail.com (L.Š.); 2 Institute of Biochemistry, Faculty of Medicine, University of Priština in Kosovska Mitrovica, 38220 Kosovska Mitrovica, Serbia; ilija.dragojevic@med.pr.ac.rs; 3 Department of Preventive Medicine, Faculty of Medicine, University of Priština in Kosovska Mitrovica, 38220 Kosovska Mitrovica, Serbia; aleksandra.ilic@med.pr.ac.rs; 4 Centre for Mental Health Protection, University Clinical Centre Niš, 18000 Niš, Serbia; nikola.st90@yahoo.com; 5 Faculty of Medicine, University of East Sarajevo, 73300 Foča, Bosnia and Herzegovina; sladojedragana@gmail.com; 6 Clinic for Dental Medicine, Faculty of Medicine, University of Niš, 18000 Niš, Serbia; gordana.filipovic@medfak.ni.ac.rs (G.F.); milena.kostic@medfak.ni.ac.rs (M.K.); 7 Clinic for Dentistry, Military Medical Academy, 11000 Belgrade, Serbia; zdenkastojanovic11167@gmail.com

**Keywords:** low-level laser therapy, wound healing, diabetes mellitus, oxidative stress, mouth mucosa

## Abstract

*Background and Objectives*: Diabetes mellitus (DM) is associated with impaired wound healing and increased oxidative stress, posing a significant challenge in dental wound healing. Low-level laser therapy (LLLT) has emerged as a potential regenerative treatment to enhance tissue repair. This study aims to investigate the effects of LLLT on oral mucosal wound healing and oxidative stress markers in rats with DM. *Materials and Methods*: Male Wistar rats (n = 108) were divided into six equal groups (healthy and diabetic, with or without mucosal ulcers, with or without LLLT). DM was induced with alloxan, and standardized mucosal ulcers were created. Every other day for 10 days, LLLT (6 J/cm^2^) was applied, and tissue samples were collected after 3, 7, and 10 days. Wound healing was assessed by planimetry, while systemic biochemical analyses included total oxidative status (TOS), total antioxidant capacity (TAC), superoxide dismutase (SOD) activity, and oxidative stress index (OSI). *Results*: LLLT significantly accelerated oral ulcer closure and showed between-group differences in redox markers. In healthy rats, LLLT increased wound closure on day 7 (*p* = 0.018). In diabetic rats, LLLT improved closure on day 3 (*p* = 0.035) and on day 7 (*p* = 0.001). Across groups, oxidative markers differed significantly (e.g., TOS on day 10 overall, *p* = 0.011; OSI on day 10 overall, *p* = 0.047; SOD *p* < 0.001 at all time points). In diabetic rats, on day 10, median TOS was lower with LLLT (*p* = 0.004). *Conclusions*: LLLT enhances oral wound healing and restores redox balance in diabetic rats, which supports the potential usage of LLLT as an adjunctive therapy for managing oral lesions in diabetic patients.

## 1. Introduction

Diabetes mellitus (DM) is a metabolic disorder characterized by chronic hyperglycemia resulting from impaired insulin secretion, impaired insulin action, or a combination of both [1]. This systemic disorder causes disturbance in carbohydrate, fat, and protein metabolism. Chronic hyperglycemia leads to damage of various structures, including small and large blood vessels, peripheral nerves, and basement membranes in different tissues, resulting in numerous acute and chronic complications [2,3]. A wide range of clinical manifestations and subjective symptoms are observed in the oral cavity of individuals with diabetes. These patients exhibit a higher prevalence of periodontitis, burning mouth syndrome, and dental caries, along with altered salivary flow and increased susceptibility to opportunistic infections [4,5]. The predisposition to oral infections is primarily attributed to reduced salivary secretion, the diminished buffering capacity of saliva, and poor oral hygiene [6]. Additionally, in these individuals, there is an increased incidence of denture stomatitis, oral lichen planus, fissured tongue, and angular cheilitis [7]. Chronic hyperglycemia contributes to structural alterations of oral tissues and impairs wound healing through associated microvascular and macrovascular complications [8,9]. These changes present significant challenges in the fabrication and wearing of removable dental prostheses. Poor adaptation or improper prosthetic design may lead to mucosal microtrauma, erosion, and lesion aggravation, ultimately resulting in chronic non-healing wounds [10,11]. Given their weakened immune response and impaired tissue repair, patients with DM require particularly careful therapeutic planning when undergoing prosthetic rehabilitation [12,13].

Impaired wound healing is among the most significant clinical challenges in patients with DM. Hyperglycemia contributes to delayed healing by increasing the accumulation of advanced glycation end products (AGEs) [14]. These AGEs, through interaction with their receptor (RAGE), induce oxidative stress, vascular inflammation, and the progression of macrovascular complications [15]. Overexpression of RAGE has been associated with delayed oral mucosal healing, likely due to reduced collagen deposition mediated by impaired fibroblast function [16,17]. Oxidative stress in DM results from an imbalance between the increased production of reactive oxygen species (ROS) and a compromised antioxidant defense system. Several mechanisms underlie this imbalance, including glucose autooxidation, nicotinamide adenine dinucleotide phosphate (NADPH) depletion via the polyol pathway, and the non-enzymatic glycation of proteins [18]. The most prominent sources of ROS under hyperglycemic conditions include mitochondrial respiration, NAD(P)H oxidase, aldehyde oxidase, xanthine oxidase, and glucose oxidase [19]. These processes contribute to tissue damage, inflammation, circulatory disruption, and delayed wound repair [20].

Low-level laser therapy (LLLT) has gained increasing attention as an effective therapeutic modality in dentistry, particularly for the management of soft tissue lesions, post-extraction healing, and prosthetic trauma [21,22,23,24]. LLLT exerts its effects by enhancing cellular metabolism, promoting mitosis, stimulating angiogenesis, improving microcirculation, and increasing collagen synthesis [25]. LLLT is thought to work through photobiomodulation, i.e., light is absorbed by mitochondrial chromophores such as cytochrome c oxidase, thus increasing ATP production and modulating ROS production, which further supports anti-inflammatory and regenerative processes [26,27,28,29]. In addition to these primary photochemical and photoenergetic effects, LLLT also induces secondary responses, including analgesia, edema reduction, inflammation modulation, and overall tissue biostimulation [30].

However, despite these benefits, evidence regarding the effects of LLLT on oral mucosal ulcers in the context of diabetes remains scarce. The intrinsic limitations of diabetic patients—including impaired angiogenesis, reduced collagen deposition, altered fibroblast and keratinocyte function, and compromised immune responses—make oral wound healing particularly challenging [8,9,10,11,12,13,14,15,16,17,18,19,20]. While most previous studies have focused on cutaneous wounds or periodontal lesions, the application of LLLT in oral ulcers among diabetic patients has not been adequately explored, and its redox-related mechanisms remain poorly understood. This gap highlights the need for experimental studies that specifically investigate whether LLLT can overcome the impaired healing environment in diabetes. Therefore, the aim of this study was to evaluate the effects of LLLT on oral mucosal wound healing and systemic markers of oxidative stress in a diabetic rat model.

## 2. Materials and Methods

### 2.1. Animals and Housing

**Figure 1:**
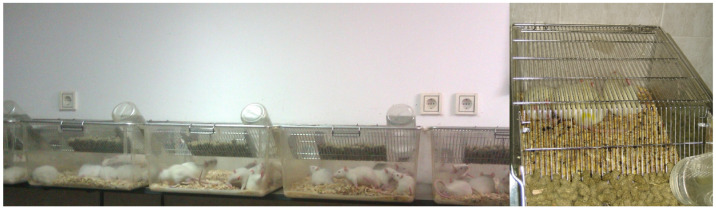
Conditions of housing and maintenance of animals during the experimental procedure.

Adult male Wistar rats weighing 260–330 g were used in this study. Animals were randomly selected from litters obtained from the Military Medical Academy, University of Defence, Belgrade. During the experiment, they were housed in the Vivarium of the Faculty of Medicine, University of Niš, under standardized conditions with 12 h light/dark cycle, temperature of 21–23 °C, and *ad libitum* access to food and water throughout the experiment. Animals were housed in cages with six animals per cage (Figure 1).

This study was approved by the Ethics Committee of the Faculty of Medicine in Priština (No. 05-1594) and the Ethics Committee of the Faculty of Medicine, University of Niš (No. 01-2625-1), obtained on 8 April 2014. The experiments were conducted at the Faculty of Medicine, University of Niš, and at the Institute of Biochemistry, Faculty of Medicine in Priština. All procedures adhered to the ethical standards of the Declaration of Helsinki, the European Convention for the Protection of Vertebrate Animals Used for Experimental and Other Scientific Purposes (CoE—ETS 123), and the Animal Welfare Law of the Republic of Serbia (Official Gazette of RS, No. 41/2009).

### 2.2. Experimental Protocol

This study was conducted in three experimental phases.

In Phase 1, animals were weighed, and fasting blood glucose levels were measured after 12 h of fasting. Blood was sampled from a tail vein, and glucose levels were measured using a glucometer (Accu-Chek Performa, Roche Diagnostics, Mannheim, Germany). Diabetes was induced by a single intraperitoneal injection of alloxan monohydrate (150 mg/kg body weight; Abcam Inc., Waltham, MA, USA) dissolved in sterile saline (NaCl 0.9%). To prevent fatal hypoglycemia, animals received a 10% glucose solution instead of drinking water for 24 h post-injection, after which they resumed normal water intake. Seventy-two hours later, glucose levels were remeasured. Rats with blood glucose > 11.1 mmol/L (200 mg/dL) were considered diabetic.

Animals were randomly allocated into six experimental groups, each consisting of 18 rats, as given in Table 1. The groups were housed separately and monitored under the same housing conditions. Sample size was based on previous similar studies with adequate power to detect biologically relevant differences, although no formal a priori calculation was performed [21]. The sample size of 18 animals per group and the sacrifice time points of days 3, 7, and 10 were selected in accordance with feasibility and established precedent in experimental oral wound-healing studies, where comparable group sizes and these canonical phases of healing (inflammatory, proliferative, and early remodeling) are typically investigated [31,32,33,34,35,36]. To control for potential systemic metabolic influences unrelated to wound healing, both healthy and diabetic non-ulcerated groups (I and IV) were included, allowing distinction of systemic metabolic effects from those specifically associated with mucosal ulceration and laser treatment.

**Figure 2:**
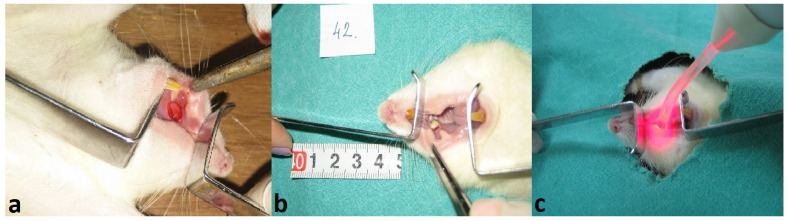
Photographs taken during the experimental procedure: (**a**) immediately post-excision; (**b**) wound imaging for planimetry; (**c**) during LLLT application.

In Phase 2, performed four weeks after diabetes induction, animals were reweighed, and glycemia was reassessed. Standardized buccal mucosal ulcers (6 mm in diameter) were created under general anesthesia (Ketamidor, Richter Pharma AG, Wels, Austria) as full-thickness excisions down to the muscle layer, ensuring consistent wound depth across all animals [31]. Mucosal disinfection was achieved using 0.12% chlorhexidine. Tissue was excised using a no. 15 scalpel blade. Hemostasis was achieved with sterile gauze. Photographs were taken immediately post-excision and on the day of sacrifice for wound area analysis using ImageJ 1.48 (NIH, Bethesda, MD, USA), with a millimeter scale included to minimize measurement error (Figure 2). Wound-healing percentage was calculated following the formula [37,38]

((wound area on day 0 − wound area on day X)/wound area on day 0) × 100%

where X is the day of animal sacrifice.

Laser-treated animals (Groups III and VI) received LLLT (630–660 nm wavelength, 7 mW output power; Scorpion Dental Optima, Sofia, Bulgaria) using a 0.196 cm^2^ probe at 6 J/cm^2^ in continuous mode, applied every other day for 10 days. The protocol of 6 J/cm^2^ applied every other day for 10 days was selected based on previous studies demonstrating effective wound healing in diabetic rodent models using comparable dosages and treatment schedules [21,37,38,39]. The irradiation time was calculated to be 168 s per session. Treatment was performed at 1 mm distance from the wound surface using a specially designed holder that ensured non-contact application and maintained a constant probe–tissue distance throughout irradiation.

In Phase 3, animals were sacrificed on days 3, 7, and 10 after the initiation of the second phase. At each time point, six animals per group were sacrificed. Afterward, blood samples were collected for biochemical analyses by cardiac puncture. Experimental animals from Groups I and IV, representing healthy and diabetic animals, respectively, served as control groups.

### 2.3. Biochemical Measurements

Blood collected in EDTA-coated tubes (Venosafe^®^, Terumo Europe, Leuven, Belgium) was centrifuged at 2500 rpm for 10 min at 4 °C, and plasma was stored at −20 °C until analysis.

The determination of total oxidative status (TOS) was based on the ability of oxidants in the sample to oxidize ferrous ions (Fe^2+^) in complex with o-dianisidine into ferric ions (Fe^3+^). In an acidic environment, ferric ions form a colored complex with xylenol orange [40]. The assay was calibrated with hydrogen peroxide, and the results were expressed in μmol of H_2_O_2_ equivalents per liter (μmol H_2_O_2_ equivalents/L). The intra-assay and inter-assay coefficients of variation for TOS were 3.7% and 5.5%, respectively.

Total antioxidant capacity (TAC) was measured by ferric reducing ability of plasma (FRAP). The FRAP assay is a colorimetric method used to assess the radical scavenging ability of antioxidants [41]. The assay was calibrated with FeSO_4_, and the results were expressed in μmol of FeSO_4_ equivalents/L. The intra-assay and inter-assay coefficients of variation for TAC were 1.8% and 4.6%, respectively.

The oxidative stress index (OSI) was calculated from the TOS and TAC values according to the following formula: OSI = (TOS/TAC) × 100. The results are expressed in arbitrary units.

The activity of superoxide dismutase (SOD) (SOD; EC 1.15.1.1) was determined spectrophotometrically according to the method of Misra and Fridovich [42]. This method is based on the inhibition of adrenochrome formation during the spontaneous oxidation of adrenaline in an alkaline medium. One unit of SOD activity (U) is defined as the amount of enzyme that inhibits adrenaline autoxidation by 50% per minute under the given conditions. SOD activity is expressed in kU/L of plasma. The intra-assay and inter-assay coefficients of variation for SOD were 7.4% and 12.9%, respectively.

### 2.4. Statistical Analysis

Data are given as continuous (measures of central tendency (mean and median), measures of variability (standard deviation), minimum (Min) and maximum (Max) values) and categorical variables, both presented as either absolute or relative frequencies. For each variable, the normality of data distribution was assessed using the Shapiro–Wilk test, and the homogeneity of variances was assessed using Levene’s test. To test hypotheses, the Student’s *t*-test for independent samples was used to compare two groups, while one-way analysis of variance (ANOVA) with Tukey’s post hoc test was used for comparisons among three or more groups. For ordinal variables and numerical variables that did not meet the assumptions of normal distribution and homogeneity of variances, non-parametric methods were used: the Mann–Whitney U test (rank-sum test) for comparisons between two groups, and the Kruskal–Wallis test for comparisons among three or more groups. Statistical hypotheses were tested at significance levels (alpha) of 0.01 and 0.05. The statistical analysis was performed using the SPSS Statistics 22 software package (SPSS Inc., Chicago, IL, USA).

### 2.5. Blinding

The investigator performing the wound planimetry and biochemical analyses was blinded to group allocation. Personnel administering laser therapy were aware of group allocation; however, they were not involved in outcome measurements or data analysis.

## 3. Results

### 3.1. Body Weight and Glycemia

Body weight was measured at three time points. As shown in Table 2, no significant difference was observed between healthy and diabetic rats at baseline. However, by the second and third measurements, diabetic animals exhibited significantly lower body weights.

Glycemia was also measured at the same time points when the animals’ body weight was measured. Initially, the values were comparable across groups. By 72 h post-alloxan injection and four weeks later, glycemia in the diabetic animals was markedly elevated (>28.2 mmol/L) compared to the healthy controls, confirming the success of diabetes induction.

### 3.2. The Percentage of Wound Healing

The percentage of wound healing (reduction in size) differed significantly between the groups where the oral mucosa defect was made. This was obvious in the experimental animals at all three measuring points—the third, seventh, and tenth day post-wounding (Table 3).

For the experimental animals sacrificed on day 3, the percentage of wound healing was significantly lower in Group V compared to Group II (*p* = 0.023), Group III (*p* < 0.001), and Group VI (*p* = 0.035).

For the experimental animals sacrificed on day 7, the most pronounced degree of wound healing was observed in Group III, with a significantly higher percentage compared to all other observed groups: Group II (*p* = 0.018), Group V (*p* < 0.001), and Group VI (*p* = 0.048). The degree of wound healing was also significantly higher in Groups II (*p* = 0.003) and VI (*p* = 0.001) compared to Group V, which had the lowest wound-healing percentage, only 60.7%.

In the experimental animals sacrificed on day 10, the percentage of wound healing was significantly higher in Group III compared to Group V of the experimental animals (*p* = 0.001). In Group III, wound healing was complete.

### 3.3. Biochemical Parameters

The results of the measured biochemical parameters are presented in Table 4.

The values of all measured parameters—TOS, TAC, OSI, and SOD—differed significantly between the experimental groups at all three measuring time points.

#### 3.3.1. TOS

For the experimental animals sacrificed on day 3, the TOS values were significantly lower in Group I compared to Group V (*p* = 0.004), as well as in Group VI compared to all other experimental groups (*p* = 0.003).

For the experimental animals sacrificed on day 7, the TOS values were significantly higher in Group VI compared to Groups I (*p* = 0.009), II (*p* = 0.004), III (*p* = 0.003), IV (*p* = 0.024), and V (*p* = 0.044) and were also higher in Group III compared to Groups II (*p* = 0.049) and V (*p* = 0.050).

For the experimental animals sacrificed on day 10, the TOS values were significantly higher in Group I compared to Groups II (*p* = 0.004) and VI (*p* = 0.009), as well as in Group V compared to the same Groups II (*p* = 0.015) and VI (*p* = 0.004).

#### 3.3.2. TAC

For the experimental animals sacrificed on day 3, the TAC values were significantly higher in Group VI compared to all other groups—I (*p* = 0.024), II (*p* = 0.006), III (*p* = 0.004), IV (*p* = 0.004), and V (*p* = 0.016)—and also in Group II compared to Group IV (*p* = 0.037).

For the experimental animals sacrificed on day 7, the TAC values in Group VI were significantly lower than those in Groups I (*p* = 0.024), II (*p* = 0.004), and V (*p* = 0.024), while Group II had significantly higher values compared to Groups III and IV (both *p* = 0.037).

For the experimental animals sacrificed on day 10, the TAC values were significantly lower in Group VI compared to Groups II and V (both *p* = 0.004).

#### 3.3.3. OSI

For the experimental animals sacrificed on day 3, the OSI values were significantly lower in Group II compared to Group III and in Group VI compared to all other groups (all *p* = 0.004).

In the experimental animals sacrificed on day 7, the OSI values were significantly higher in Group VI compared to Groups I (*p* = 0.010), II (*p* = 0.004), III (*p* = 0.025), IV (*p* = 0.025), and V (*p* = 0.004), while the values in Group II were significantly lower than those in Group III (*p* = 0.004).

For the experimental animals sacrificed on day 10, the OSI values were significantly lower in Group II compared to all other groups: I (*p* = 0.004), III (*p* = 0.037), IV (*p* =0.03), V (*p* = 0.04), and VI (*p* = 0.044).

#### 3.3.4. SOD Activity

For the experimental animals sacrificed on day 3, the SOD values were significantly higher in Group I compared to Groups III (*p* = 0.004), IV (*p* = 0.006), V (*p* = 0.004), and VI (*p* = 0.004). The values were also higher in Group II compared to Groups III (*p* = 0.003), IV (*p* = 0.006), V (*p* = 0.003), and VI (*p* = 0.003). Group III had higher values than Groups V (*p* = 0.010) and VI (*p* = 0.004), and Group IV had significantly higher values than Groups III (*p* = 0.010), V (*p* = 0.004), and VI (*p* = 0.004).

For the experimental animals sacrificed on day 7, the SOD values were significantly higher in Group I compared to all other groups: II (*p* = 0.003), III (*p* = 0.003), IV (*p* = 0.006), V (*p* = 0.003), and VI (*p* = 0.004). Group II’s values were significantly higher than those in Groups V and VI (both *p* = 0.003). Similarly, Group III’s values were higher than those in V and VI (both *p* = 0.003), and Group IV had significantly higher values than Groups V (*p* = 0.003) and VI (*p* = 0.004).

For the experimental animals sacrificed on day 10, the SOD values were significantly higher in Group I compared to all other groups: II (*p* = 0.004), III (*p* = 0.004), IV (*p* = 0.010), V (*p* = 0.004), and VI (*p* = 0.004). Group II’s values were significantly higher than those in Groups V and VI (both *p* = 0.004), as were the values in Group III (both *p* = 0.004) and Group IV (both *p* = 0.004). The SOD values were also significantly higher in Group V compared to Group VI (*p* = 0.050).

**Figure 3:**
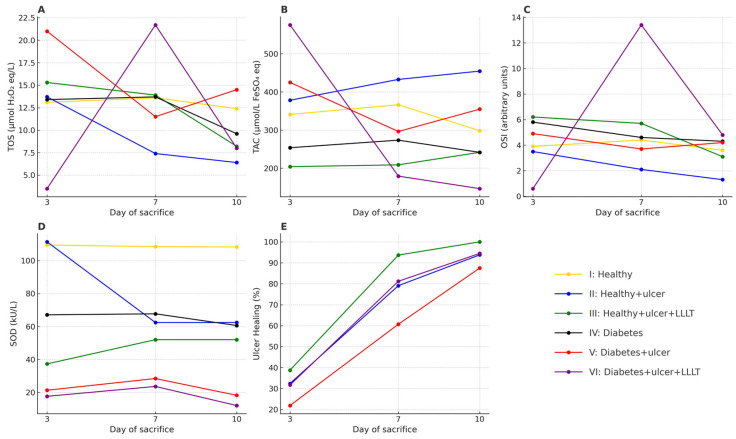
Changes in the monitored parameters in experimental animals. Changes in TOS (**A**), TAC (**B**), OSI (**C**), SOD activity (**D**), and ulcer healing (**E**) on days 3, 7, and 10. TOS—total oxidative status; TAC—total antioxidant capacity; OSI—oxidative stress index (arbitrary units); SOD—superoxide dismutase activity.

Figure 3 summarizes the observed effects of LLLT on oral mucosal wound healing and systemic oxidative stress markers across six experimental rat groups over three time points: day 3, 7, and 10 after ulcer induction. This figure provides a comprehensive visual summary of both healing outcomes and oxidative stress modulation by LLLT, highlighting its therapeutic efficacy, especially in diabetic conditions.

## 4. Discussion

LLLT has emerged as a noninvasive modality to promote tissue regeneration, modulate inflammation, and accelerate chronic wound healing [43]. At the cellular level, LLLT enhances the mechanisms essential for tissue repair [44]. Several studies have confirmed the beneficial effects of LLLT on oral wound healing, even in pathological conditions such as DM [45]. Diabetes predisposes patients to oral mucosal erosion and ulceration, particularly at the interface with dental prostheses [10]. Delayed wound healing in individuals with DM not only causes discomfort and a higher risk of infection but also compromises prosthetic outcomes, often necessitating repeated treatment and the redesign of restorations.

In the present study, animals injected with alloxan developed persistent hyperglycemia (>28.2 mmol/L) and significant body weight loss, consistent with previously validated models of chemically induced type 1 diabetes [46,47]. These systemic changes reflected a catabolic metabolic state that impaired tissue repair mechanisms [48]. Our findings further demonstrate that diabetes significantly delays wound healing and disrupts systemic redox homeostasis, as evidenced by increased TOS and OSI along with decreased TAC and SOD activity (Table 3). Importantly, the inclusion of non-ulcerated healthy (Group I) and diabetic (Group IV) controls enabled us to account for systemic metabolic effects, ensuring that the observed differences primarily reflect wound-related and treatment-specific responses. These results align with the well-established role of hyperglycemia in impeding normal healing [49,50]. Diabetic rats with mucosal ulcers showed severely delayed healing (by contrast, healthy rats achieved near-complete healing by day 10) and elevated oxidative stress markers, consistent with literature suggesting the effects of hyperglycemia [51].

The LLLT-treated diabetic animals exhibited significantly improved wound healing, with closure rates comparable to non-diabetic controls by day 10. These findings support previous studies that show that LLLT can promote mechanisms of wound healing even under metabolic stress [52,53]. Because photobiomodulation was delivered repeatedly, its effects are cumulative and time-dependent rather than attributable to a single exposure. In our data, the largest incremental gains in wound closure with LLLT versus non-LLLT were observed on day 7, in both health states. By day 10, the between-group gap narrowed in the healthy animals, while a clinically relevant advantage persisted in the diabetic animals. This pattern suggests a biphasic time course in which the LLLT benefit is most pronounced around the transition from inflammation to proliferation (day 7), with diminishing marginal returns as wounds approach closure (day 10).

TOS reflects the cumulative burden of oxidants such as ROS, lipid peroxides, and organic hydroperoxides [40], while TAC indicates the plasma’s total non-enzymatic antioxidant potential [41], whereas OSI, a ratio of TOS to TAC, quantifies redox imbalance. Redox markers mirror wound-closure nonlinear behavior. In laser-treated diabetic rats (VI), we observed an early antioxidant shift on day 3, with TOS markedly lower and TAC higher, resulting in an extremely low OSI. By day 7, however, VI exhibited a transient oxidative rebound with the highest TOS and highest OSI among all groups, before normalizing by day 10. This trajectory is consistent with a hormetic/biphasic LLLT response in metabolically stressed tissue—an early suppression of oxidative burden followed by a mid-phase rise that likely reflects increased cellular activity and ROS signaling during robust granulation and matrix turnover and subsequent partial resolution as healing progresses.

These effects were most notable on day 3, during the inflammatory phase of healing, indicating that the early initiation of LLLT plays a critical role in restoring redox homeostasis. A similar trend was observed in a recent animal study where LLLT doses of 4–8 J/cm^2^ enhanced healing and modulated oxidative biomarkers in diabetic wounds [54]. Cellular studies further explain the mechanisms by which LLLT in diabetic fibroblasts suppresses ROS production via FOXO1 inhibition [55].

SOD activity exhibits a distinct pattern. Across time points, the LLLT-treated groups showed lower plasma SOD than their non-LLLT counterparts within the same health state, and diabetic groups were uniformly lower than the healthy groups. One parsimonious interpretation is reduced upstream superoxide generation in laser-treated animals (with a reduced inducible requirement for circulating SOD) alongside redistribution to healing tissues. Regardless, this consistent directionality (III < II; VI < V at each time) underscores that plasma SOD does not track with gross healing in a linear fashion and should not be used alone as a surrogate for LLLT benefit. The combined OSI (integrating TOS/TAC) aligns more closely with the observed healing kinetics. The present results align with reports demonstrating that red and infrared LLLT reduces lipid peroxidation and boosts antioxidant enzymes such as SOD and catalase in diabetic cutaneous wounds [56,57]. Healthy laser-treated rats also showed modest increases in TAC and SOD on day 10 (Table 3 and Figure 3), which suggests a general mitochondrial upregulation and regenerative stimulus [58,59]. These findings reflect broader LLLT-induced metabolic enhancements [60].

Taken together, our results support a nonlinear, group-specific dose–time relationship: an early (day 3) antioxidant shift, most pronounced in diabetic ulcers, with parallel, modest gains in closure; a mid-phase (day 7) oxidative peak, again, the greatest in diabetes, coinciding with the largest healing advantage versus non-LLLT; and a late (day 10) convergence of redox indices with persistent healing benefit in diabetes. This time-structured response calls for dose optimization (energy density and/or session spacing) tailored to metabolic status, potentially moderating cumulative exposure around day 7 in diabetes while preserving early treatments that appear decisive for setting a pro-healing redox trajectory.

Our design sampled independent cohorts at days 3, 7, and 10; thus, the above interactions were evaluated by between-group comparisons within each time point. A factorial framework (group × time) or rank-based two-way methods could formally test interaction effects, but even within the present analysis, the direction and timing of the changes were consistent across markers and outcomes, which strengthens the inference of a biphasic, context-dependent response to repeated 6 J/cm^2^ exposure.

Importantly, the most pronounced differences in oxidative parameters between the laser-treated and untreated diabetic animals occurred during early wound healing (days 3 and 7). The magnitude of LLLT’s redox effects depends on the metabolic context. On day 3, the antioxidant swing with LLLT was far larger in diabetic ulcers (VI) than in healthy ulcers (III)—VI achieves the lowest OSI across all groups, whereas III remained intermediate. Conversely, on day 7, the oxidative “overshoot” was, again, most evident in VI, not III. These contrasts indicate that the baseline redox imbalance in diabetes amplifies both the early beneficial shift and the mid-phase rebound, i.e., a steeper dose–time curve in diabetes. Clinically, this implies that diabetic wounds may need tighter dose spacing or titration around the day-5–8 window to blunt the day-7 spike while preserving the early gain. By day 10, oxidative stress markers had begun to normalize, indicating that repeated LLLT fosters a progressive reestablishment of redox balance. This pattern is consistent with the biphasic oxidative response observed during the tissue-healing process [20,61]. The results reinforce the reproducibility and efficacy of using a standardized LLLT protocol (6 J/cm^2^ every other day for 10 days). While previous studies have shown variable results depending on laser wavelength and energy density [62,63], the findings of the present study demonstrate consistent improvement in both healing and redox regulation using a uniform laser protocol.

Finally, the limitations of this study include the absence of histological analysis of wound tissues and the lack of molecular profiling of oxidative signaling pathways. Additionally, the model included only male rats, limiting insights into potential sex-related differences in healing responses. Future studies might focus on examining morphological changes in the wound-healing process and determining the type of cells and molecular marker expression influenced by LLLT.

## 5. Conclusions

This study reveals and corroborates that DM markedly impairs oral mucosal wound healing and disrupts systemic redox homeostasis, as evidenced by elevated oxidative stress markers (TOS, OSI) and diminished antioxidant defense (TAC, SOD). These alterations significantly delay tissue repair and complicate clinical outcomes, particularly in the context of prosthetic rehabilitation. In this experimental model, LLLT demonstrated a beneficial effect on systemic oxidative stress markers and accelerated oral wound healing, particularly in diabetic rats. Specifically, it reduced TOS and OSI while increasing TAC and SOD activity. These effects were most pronounced when treatment was initiated early and applied consistently during the healing process. These findings provide preclinical evidence that LLLT can modulate redox balance and enhance healing dynamics in metabolically compromised tissue. While the data are promising, they should be interpreted as hypothesis-generating rather than immediately translatable to practice. Given the absence of histological assessment and human validation in this study, further research is warranted, specifically, histopathological confirmation in animal models and controlled clinical trials, to substantiate and extend our observations. Thus, our results suggest that LLLT holds translational potential for wound management in diabetes, but firm clinical recommendations await additional confirmatory studies.

## Figures and Tables

**Table 1:** Distribution of experimental animals by group.

Group	Number of Animals	General Condition	Ulceration	Treatment
I	18	Healthy	No	None (control)
II	18	Healthy	Yes	No treatment (spontaneous healing)
III	18	Healthy	Yes	LLLT
IV	18	DM	No	None (control)
V	18	DM	Yes	No treatment (spontaneous healing)
VI	18	DM	Yes	LLLT

Groups of adult male Wistar rats (n = 108) divided according to health status, ulcer presence, and treatment. DM—diabetes mellitus; LLLT—low-level laser therapy.

**Table 2:** Body weight and glycemia of healthy animals and animals with induced diabetes.

Measurement Time	Groups	Mean ± SD	*p* Value
**Body Weight (g)**			
First	Healthy	300.7 ± 24.3	0.179
	DM	306.8 ± 22.6	
Second	Healthy	319.6 ± 24.4	<0.001
	DM	287.2 ± 34.8	
Third	Healthy	444.1 ± 35.4	<0.001
	DM	272.2 ± 51.8	
**Glycemia (mmol/L)**			
First	Healthy	5.7 ± 0.4	0.530
	DM	5.6 ± 0.5	
Second	Healthy	5.7 ± 0.3	<0.001
	DM	28.3 ± 3.1	
Third	Healthy	5.7 ± 0.4	<0.001
	DM	28.2 ± 3.5	

Data are presented as mean ± standard deviation (SD) and median (Q1–Q3). Measurement times: first—prior to alloxan administration; second—72 h after alloxan injection; third—four weeks after alloxan administration. DM—diabetes mellitus. *p*-values were obtained using Student’s *t*-test.

**Table 3:** Percentage of ulcer healing by group in relation to the day of sacrifice.

Groups	Ulcer Healing (%)		*p* Value
	n	Mean ± SD	
**3rd Day of Sacrifice**			
II	6	32.4 ± 4.3	
III	6	38.8 ± 6.2	*p* = 0.001
V	6	21.9 ± 7.3	
VI	6	31.7 ± 4.2	
**7th Day of Sacrifice**			
II	6	79.1 ± 7.8	
III	6	93.7 ± 3.8	*p* < 0.001
V	6	60.7 ± 10.7	
VI	6	81.2 ± 7.0	
**10th Day of Sacrifice**			
II	6	93.8 ± 7.6	
III	6	100.0 ± 0.0	
V	6	87.5 ± 4.7	*p* = 0.002
VI	6	94.5 ± 3.3	

Data are presented as mean ± standard deviation (SD). Group II: healthy + ulcer (no treatment); Group III: healthy + ulcer + LLLT; Group V: DM + ulcer (no treatment); Group VI: DM + ulcer + LLLT. LLLT—low-level laser therapy; DM—diabetes mellitus. *p*-values were based on one-way analysis of variance (ANOVA).

**Table 4:** TOS, TAC, OSI, and SOD activity in plasma of experimental animals at all three sacrifice time points.

Groups	Day of Sacrifice		
	3rd	7th	10th
**TOS (μmol H_2_O_2_ equivalents/L)**			
I	13.1 (10.0–14.7)	13.6 (10.0–17.4)	12.4 (8.6–17.4)
II	13.7 (9.4–17.9)	7.4 (5.4–12.8)	6.4 (3.5–7.5)
III	15.3 (12.6–17.7)	13.9 (11.8–16.1)	8.2 (5.6–13.7)
IV	13.4 (6.2–20.6)	13.7 (6.2–20.6)	9.6 (6.3–17.2)
V	21.0 (16.9–28.3)	11.5 (9.4–12.3)	14.5 (10.7–21.2)
VI	3.5 (1.3–3.5)	21.7 (17.4–30.8)	8.0 (5.4–8.3)
*p* value	*p* = 0.001	*p* = 0.004	*p* = 0.011
**TAC (μmol/L FeSO_4_ equivalents)**			
I	341.1 (201.2–469.3)	366.5 (201.2–430.9)	298.4 (201.2–472.7)
II	378.3 (329.9–405.2)	432.9 (299.5–535.0)	454.7 (316.2–496.0)
III	204.3 (187.2–340.8)	209.0 (160.6–343.6)	241.8 (179.4–449.2)
IV	253.9 (206.7–318.2)	273.7 (180.2–356.8)	241.8 (195.2–424.2)
V	425.1 (314.3–535.0)	296.4 (232.8–433.6)	354.9 (346.3–523.8)
VI	575.7 (541.0–756.6)	179.4 (107.6–258.9)	146.7 (138.9–288.6)
*p* value	*p* = 0.004	*p* = 0.016	*p* = 0.011
**OSI**			
I	3.9 (2.7–6.4)	4.4 (2.1–6.7)	3.6 (2.7–5.4)
II	3.5 (2.7–4.7)	2.1 (1.0–3.3)	1.3 (1.0–1.9)
III	6.2 (5.2–7.9)	5.7 (4.7–8.0)	3.1 (1.5–7.2)
IV	5.8 (2.0–10.0)	4.6 (2.3–8.3)	4.3 (2.6–5.8)
V	4.9 (4.3–8.7)	3.7 (2.5–5.2)	4.2 (2.0–5.9)
VI	0.6 (0.2–0.6)	13.4 (8.1–23.8)	4.8 (2.6–5.7)
*p* value	*p* = 0.003	*p* = 0.002	*p* = 0.047
**SOD (kU/L)**			
I	109.5 (103.6–115.3)	108.5 (103.4–117.0)	108.3 (94.9–116.8)
II	111.4 (108.0–114.8)	62.4 (52.0–72.8)	62.4 (46.8–83.2)
III	37.3 (30.2–47.5)	52.0 (52.0–72.8)	52.0 (45.8–72.8)
IV	67.1 (53.3–80.8)	67.7 (41.0–91.6)	60.6 (53.2–78.1)
V	21.3 (14.2–28.4)	28.4 (14. 2- 28.4)	18.2 (14.2–27.1)
VI	17.6 (11.1–22.3)	23.6 (14.9–28.4)	12.0 (11.1–19.5)
*p* value	*p* < 0.001	*p* < 0.001	*p* < 0.001

Data are presented as median and interquartile range (Q1–Q3). Experimental groups: Group I—healthy control; Group II—healthy + ulcer, no treatment; Group III—healthy + ulcer + low-level laser therapy (LLLT); Group IV—diabetes mellitus (DM) control; Group V—DM + ulcer, no treatment; Group VI—DM + ulcer + LLLT. TOS—total oxidative status; TAC—total antioxidant capacity; OSI—oxidative stress index (arbitrary units); SOD—superoxide dismutase activity. *p*-values were based on the Kruskal–Wallis test.

## Data Availability

The data presented in this study are available on request from the corresponding author.
